# Multidisciplinary digital methodologies for documentation and preservation of immovable Archaeological heritage in the Khovd River Valley, Western Mongolia

**DOI:** 10.12688/f1000research.126740.1

**Published:** 2022-11-03

**Authors:** Michael T. Fisher, Dovydas Jurkenas, Amina Jambajantsan, Bayarsaikhan Jamsranjav, Eredene-Ochir Nasan-Ochir, Eregzen Gelegdorj, Munkhbayar Chuluunbat, Michael Petraglia, Nicole Boivin

**Affiliations:** 1 Department of Archaeology, Max Planck Institute for Geoanthropology, Jena, Thuringen, 07743, Germany; 2 National Museum of Mongolia, Ulaanbaatar, Ulaanbaatar, 14201, Mongolia; 3 Institute of Archaeology, Mongolian Academy of Sciences, Ulaanbaatar, Ulaanbaatar, 13330, Mongolia; 4 History and Social Sciences Department, Institute of Social and Human Sciences, Khovd State University, Khovd, Khovd, 84000, Mongolia; 5 Australian Research Centre for Human Evolution, Griffith University, Brisbane, Queensland, 4111, Australia; 6 School of Social Science, University of Queensland, Brisbane, Queensland, 4072, Australia; 7 Department of Anthropology, National Museum of Natural History, Smithsonian Institution, Washington, D.C., 20560, USA

**Keywords:** Mongolian archaeology, digital cultural heritage preservation, Machine Learning, remote sensing, landscape archaeology, GIS, semantic data modelling, transmethodology, Erdeneburen Dam, endangered archaeology

## Abstract

**Background:** The archaeological and ethnographic heritages of Mongolia reflect a multi-millennial continuity of typically mobile-pastoral occupations across sparsely populated, environmentally diverse landscapes, but the threats of modernisation and industrialisation to those heritages are nevertheless present and substantial. The construction of the Erdeneburen Hydroelectric Dam on the Khovd River in western Mongolia is planned to submerge hundreds of archaeological features and jeopardise at least another thousand.

**Methods:** The Mongolian Archaeology Project: Surveying the Steppes, in collaboration with the Mongolian Institute of Archaeology, integrates a variety of digital techniques including GIS (geographic information systems), Machine Learning automated site detection, drone mapping, and Structure-from-Motion LiDAR scanning to document the endangered archaeology. This paper presents the resulting dataset of archaeological features across three different impact zones associated with the dam construction and evaluates the degree of efficacy of the initial data integration strategy through informal partner feedback and self-assessment.

**Results:** While only approximately 20% of the documented sites fall within the planned flood zone, the remaining sites will be subjected to collateral threats such as industrial and infrastructural development that will necessitate extended monitoring, both temporally and spatially. In consideration of these results, this paper argues that a ‘responsive’ mode of heritage disaster intervention can bridge the gap between ‘reactive’ and ‘proactive’ modes, but requires development of an integrated (digital) methodology.

**Conclusions**: The paper concludes by offering a new, more interconnected ‘transmethodology’ that addresses spatiality, sub-sampling, data reuse, and community input across multiple disciplines such as cultural heritage preservation, salvage archaeology, computer vision, and community archaeology. The authors developed this ‘transmethodology’ and the resulting workflows out of a theoretical framework that considers principles of Symmetrical Archaeology, Resilience Humanitarianism, and the CARE standard for inclusive data management (Collective benefit, Authority to control, Responsibility, and Ethics).

## Introduction

**Figure 1.**
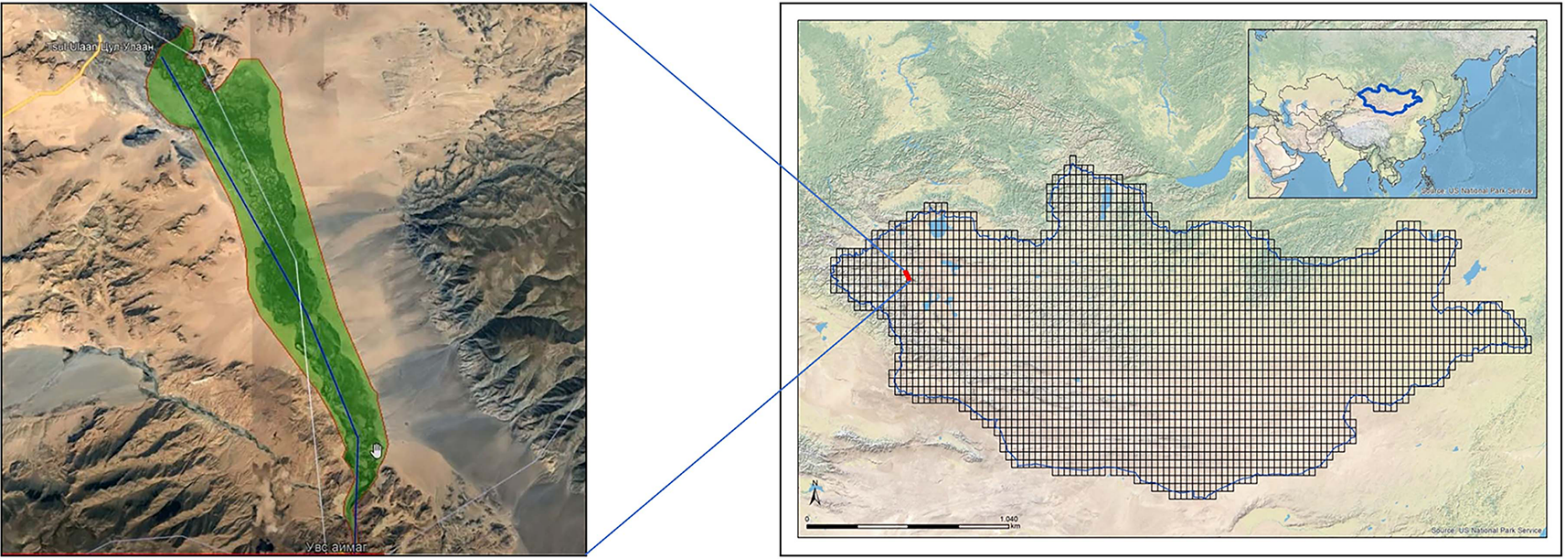
Map of Mongolia showing the MAPSS grid and location of the planned Erdeneburen Dam flood zone. Map sources (open access): Esri, Maxar, GeoEye Earthstar Geographics, cNEs/Airbus Ds, UsDA, UsGs, AeroGRID, IGN, and the GIS User Community (left); US National Park Service (right).

Digital archaeology is a rapidly emerging sub-discipline that draws on a wide range of methodologies, theoretical dispositions, and techniques to integrate traditional archaeological methods with computer applications, digital recording equipment, and, ideally, processes of digitalisation ( Richards-Rissetto & Landau 2019; Huggett 2017, 2015; Zubrow 2006). Increasingly, digital archaeology approaches are advancing the methodologies of adjacent disciplines such as cultural heritage preservation ( Lukas *et al.* 2018; MacFarland & Vokes 2016; Clarke 2015; Esteva *et al.* 2010; Hadjimitsis *et al.* 2009; Witcher 2008; Reichel 2005), expanding the potential for breadth of coverage and depth of recorded information. This is especially evident in the practices of remote survey ( VanValkenburgh & Dufton 2020; Luo *et al.* 2019; Bradley 2006), particularly for documenting archaeological sites and monitoring their threats and disturbances (e.g., Breen *et al.* 2022; Tews *et al.* 2022; Hammer *et al.* 2018; Fradley & Sheldrick 2017; Casana & Laugier 2017; Parcak *et al.* 2016; Casana & Panahipour 2014; Parcak 2007; Box 1999). This paper demonstrates an approach to integrating various digital-archaeology methods for cultural heritage preservation in Mongolia, focusing on archaeological sites affected by the planned flooding to result from the Erdeneburen Hydroelectric Dam Construction Project in the Khovd River Valley, Khovd *aimag*/province ( Figure 1).

The Mongolian Archaeology Project: Surveying the Steppes (MAPSS) is a cultural heritage preservation and archaeological research endeavour that draws on digital methodologies to detect, interpret, and assess archaeological and ethnographic sites across Mongolia, generating a large body of primarily ‘born-digital’ (Scott *et al.* 2021) data. Similar to other large-scale heritage documentation projects (e.g., Westley *et al.* 2021; Stein 2020; Green *et al.* 2019; Bewley *et al.* 2016), MAPSS deploys remote sensing methods such as interpretation of high-resolution satellite imagery to rapidly identify surficial archaeological remains, confirming and complementing those data with targeted pedestrian surveys. As a digital archaeology project, it integrates its various digital techniques through a strategic aggregational methodology and integrates its datasets in a geospatial semantic database. Its aim is to avail those data to other researchers, the general public, and, especially, Mongolian heritage professionals. These categories describe overlapping, non-exclusive group memberships, but represent different modes of data dissemination. Moreover, the theoretical bases of this research incorporate elements of Symmetrical Archaeology and Resilience Humanitarianism to essentialise both the FAIR ^1^ and CARE ^2^ data management principles by incorporating local input into the workflows and feedback systems.

Mongolia presents unique challenges to cultural heritage preservation, as it is the least densely populated nation on earth ( Cui *et al.* 2019). The rural economy predominantly comprises mobile pastoralism, with little agriculture, industry, or sedentarism to disturb its archaeological remains ( Honeychurch 2010). But sustainable, traditional economies are shifting toward more destructive, modern industrial practices nevertheless (ibid.), and the rapid growth of the capital city of Ulaanbaatar, at the expense of the rural population count ( Cui *et al.* 2019), appears to be initiating a transformation of the pastoral countryside and its perdurance of pre-modern social praxis into an industrialised hinterland supporting the urban capital.

**Figure 2.**
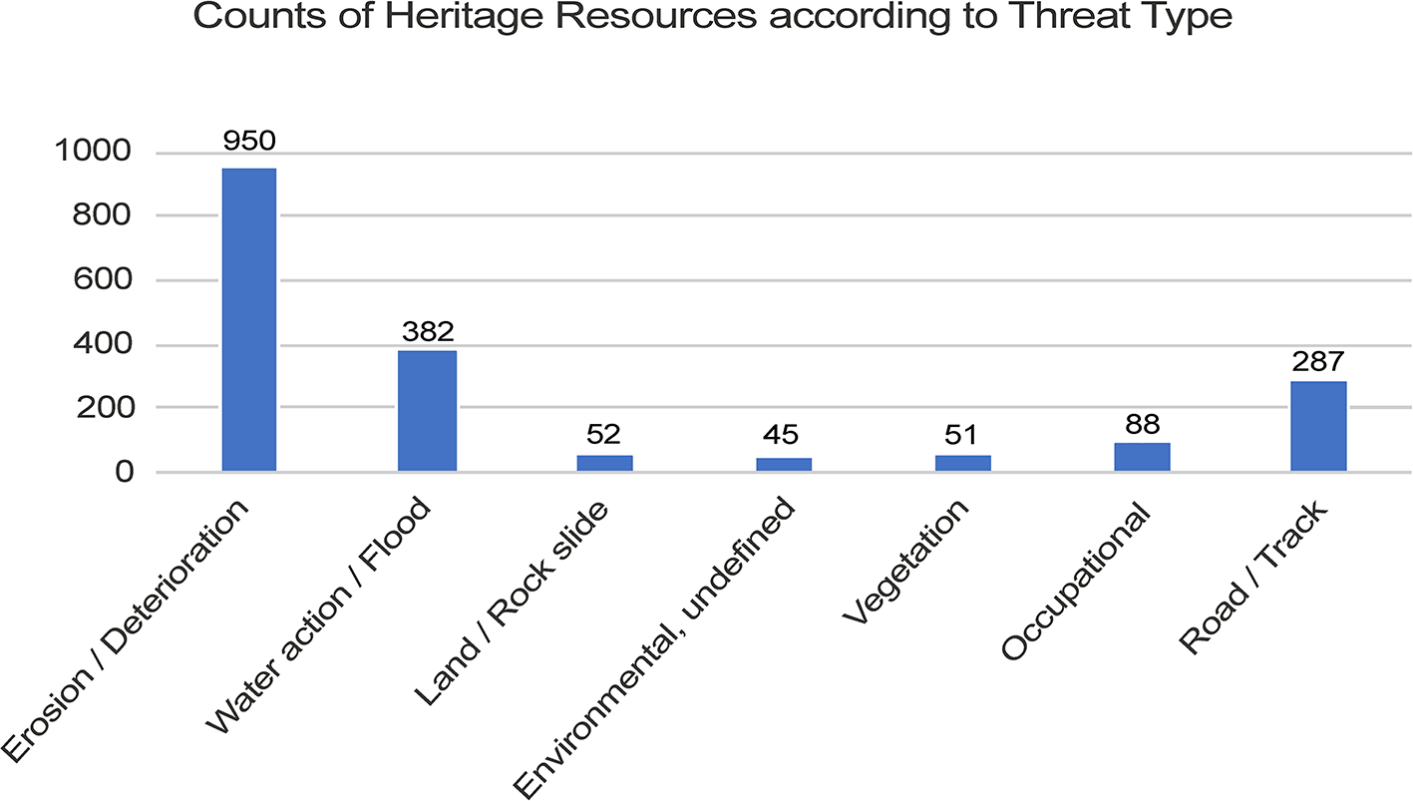
Counts of heritage resources according to threat type.

Some of the most readily identifiable threats to archaeology across the Asian and African cultural landscapes include human activities such as industrial development and agricultural and urban expansion (e.g., Ekblom *et al.* 2019; Zerbini & Sheldrick 2017; Williams 2016). Using remote sensing methods, the MAPSS project’s risk analysis of archaeological remains in Mongolia indicates that environmental processes such as wind and water activity have posed the most glaring threats there, contrastingly ( Figure 2).

With such low population density levels, this is an unsurprising observation. Threats from development and economic activities such as mining (both legal and illegal) to archaeological remains are nevertheless present, but are less visibly prominent—and so harder to detect remotely—due to their nascence. However, these practices are poised to become highly destructive to Mongolian cultural heritage in the near future. The Erdeneburen Hydroelectric Dam Construction Project illustrates this with great urgency, as it is planned to flood an approximately 3,500 km ^2^ section of the Khovd River Valley by 2027, submerging and endangering hundreds of archaeological sites alongside traditional lifeways. The national government has funded the Mongolian Institute of Archaeology to conduct emergency pedestrian survey and salvage excavation operations as the dam construction begins. The MAPSS project offered to assist with the site documentation process using digital methods in order to generate complementary datasets.

The working hypothesis of this research is that an integrative, inclusive, and attentive digital archaeology program—which features data sharing, local input in data modelling, and methodological training—can have a net positive impact on archaeological recording systems and cultural heritage conservation in Mongolia, and support ‘proactive’ heritage preservation, rather than ‘reactive’ interventions ( Faizi *et al.* 2017: 31-35). In many instances, such as the construction of the Erdeneburen Dam, a ‘responsive’ mode of intervention is warranted. In these cases, the threat event triggers the response, which should then itself pre-empt establishment of proactive heritage preservation practices going forward, rather than deploying successive ‘reactive’ interventions ( Fisher, in press). The MAPSS project realises both ‘responsive’ and ‘proactive’ modes of heritage preservation through its data and digitalisation integration strategies.

‘Responsive’ and ‘proactive’ digital solutions should adhere to both the FAIR and CARE principles, not simply because they have become universal standards in data management, but because, we suggest, an interrelationship exists between reusability, inclusivity, and sustainability. Toward these ends, the MAPSS data collection and management systems are designed to consider modelling uncertainty at scale, multilinguality, multivocality, and interpretive multiplicity. Thus, the project balances capture of metadata, paradata, resource relationships, site conditions, and archaeological interpretation with the scope, ambition, and inclusivity of populating a national cultural heritage database.

The outcomes of MAPSS’ involvement with the Erdeneburen Dam Project are both empirical and methodological. The opportunity to form a collaboration between MAPSS and the Mongolian Institute of Archaeology served as a means of using digitally integrated methods to document endangered heritage and as a test case for the project’s data transmission pipelines and integration strategies. Thus, the results of this research are presented here in terms of both the data generated and, more generally, the utility of the system design and its impact. Based on informal reporting by partners in the Mongolian Institute of Archaeology, the overall impact of the MAPSS data integration program was positive, but the reporting included constructive feedback as well, which has led to MAPSS redefining its methodology and reformulating some of its techniques and workflows. This paper will start by describing the initial MAPSS methodology and the methods it comprises, before presenting the datasets, analyses, and outcomes, which include a reimagined, pyramidal, and vertically integrated MAPSS methodology or ‘transmethodology’ ( Khwaja & Kousholt 2021; see below).

## Methods

The primary function of the MAPSS project is to document the archaeological landscapes of Mongolia through manual remote sensing (MRS) of archaeological sites and features (together called “heritage resources”) by interpreting landscape features seen on high-resolution satellite imagery, as well as through automated detection of certain heritage resource types using a specialised subset of machine learning (ML) methods called deep learning (DL). MAPSS also relies on utilising data furnished by partner institutions in Mongolia such as the National Centre for Cultural Heritage, the Institute of Archaeology, and Khovd University, and digitally integrates the resulting outputs ( Fisher 2022).

MRS of archaeological features is at the core of the project’s methodology, working at a pace of approximately 25,000 sq. km per year to identify heritage resources using freely available, open-access imagery from Google Earth, Bing, Esri, HERE WeGo, and Maxar Technologies. MAPSS analysts follow now-standard methods for satellite imagery interpretation (e.g., Fowler 2010; Hritz 2010; Ur 2006; Wilkinson *et al.* 2006; Richards 2005), marking the locations of archaeological and ethnographic features such as funerary monuments, ritual monuments, and campsites, as well as assessing disturbances and threats to heritage resources in accordance with ICCROM (International Centre for the Study of the Preservation and Restoration of Cultural Property) guidelines (see Jigyasu 2019). MAPSS employs a condition/threat assessment model based on the three-tier system for interpreting effects (disturbances), threats, and threat drivers (see Zaina 2019), previously implemented by the Endangered Archaeology in the Middle East and North Africa (EAMENA) project ( Ten Harkel & Fisher 2021) but modified further for purposes of localisation, semantic efficiency, and data reusability.

In parallel, the MAPSS project developed DL techniques using ‘supervised learning’ functions in order to identify potential archaeological features such as funerary and habitation sites in the Khovd River Valley region, akin to ‘pure prediction’ of archaeological remains ( Bevan 2015: 1478). This approach automates remote site discovery by using ArcGIS Pro’s dedicated Deep Learning Toolset, which features a third-party DL Python application programming interface (API) (TensorFlow, PyTorch, or Keras) to detect pixel clusters (‘objects’) in an image, deploying a specified Python raster function to process each object. By training the system using known datasets of heritage resources and ready-made DL models such as Mask Region-based Convolutional Neural Network (R-CNN), You Only Look Once v3 (YOLOv3), or Single Shot Detector (SSD), analysts run the selected model against an input raster image to produce results according to each object class (such as ‘burial mound’ or ‘winter-camp habitation site’). The input raster images are high resolution and freely accessible, with a typical spatial resolution of 0.6 m.

DL models rely on ‘neural networks’, or series of algorithms that work together to mirror human brain function using weights and biases for decision making ( Casini *et al*. 2021; Shrestha & Mahmood 2019). For automated object detection across large images (in geographic terms, landscapes), convolutional neural networks (CNNs) aggregate constituent networks through a sequence of typically four layers that first sort pixel values (input layer), then filter pixels to identify edges (convolutional layer), then pool values (pooling layer), and finally connect all layers and assign a unique value to each object (fully-connected layer; Shrestha & Mahmood 2019: 53044). Supervised learning is a function of DL in which a human operator uses image data to label objects in a training dataset composed of business data (ibid.: 11). It is useful for extrapolating outward from known business data to unknown business data, identifying all objects that fall within the range of pixel-value signatures per object class according to the algorithms of the DL model.

Operationally, the first steps of supervised learning are typically to obtain the open-access imagery data and normalise them by modifying each tensor so that the mean is 0 and the standard deviation is 1, allowing the neurons in the network to “learn” faster (MAPSS uses Anaconda Jupyter Notebook for normalisation). To then create training data, one makes a visual assessment of the geographical area, imports the business dataset (if available), draws bounding boxes or polygons around the known objects, and exports the polygons into a folder containing images, their labels, an ESRI-accumulated statistical JSON file, and an ESRI model-definition file. Then one chooses a DL model for training.

MAPSS tested five DL models (SSD, RetinaNet, YOLOv3, Faster R-CNN, and Mask R-CNN) using a geographically constrained subset of its geospatial dataset from western Mongolia. SSD is a model that runs once over the input image and is designed for both speed and accuracy ( Liu *et al.* 2015). The backbone of this model is typically a network such as ResNet, and the SSD head comprises one or more overlying convolutional layers. The model divides the image according to a grid and within each grid cell detects objects, predicting their class and location. RetinaNet is a one-stage object detection model ( Alhasanat *et al.* 2021), often applied to aerial and satellite imagery, and has a high true-positive rate for small-scale objects. RetinaNet also uses a ResNet as a backbone network, but uses Focal Loss as an enhancement over Cross-Entropy Loss to handle the class imbalance problem. YOLOv3 has a different backbone than the previous two as it uses Darknet-53, which employs 53 convolutional layers and so is generally faster than the ResNet family ( Redmon & Farhadi 2018). It integrates upsampling and concatenation of feature layers with earlier layers, detecting at three different scales, and so can be effective for sensing objects of varying size. Faster R-CNN relies on a convolutional algorithm called the Region Proposal Network (RPN) and a CNN backbone. RPN algorithms use low-cost region proposals to predict object boundaries and “objectness” scores for each pixel cluster ( Shaoqing *et al.* 2015).

Mask R-CNN is a variant of the Faster R-CNN model, also utilising the RPN convolutional algorithm, but it can additionally predict a segmentation mask that marks the individual pixels composing each object. Mask R-CNN has so far been the most successful model applied to MAPSS data, with a 63% success rate against the training dataset, making it the best choice for automated remote sensing detection in the Khovd River Valley region. MAPSS ran it in ArcGIS against the selected raster input and then manually evaluated the initial output, deleting false positives and adding false negatives. Finally, analysts exported the output as a.CSV file, deployable as a layer in any geographic information systems (GIS) application.

**Figure 3.**
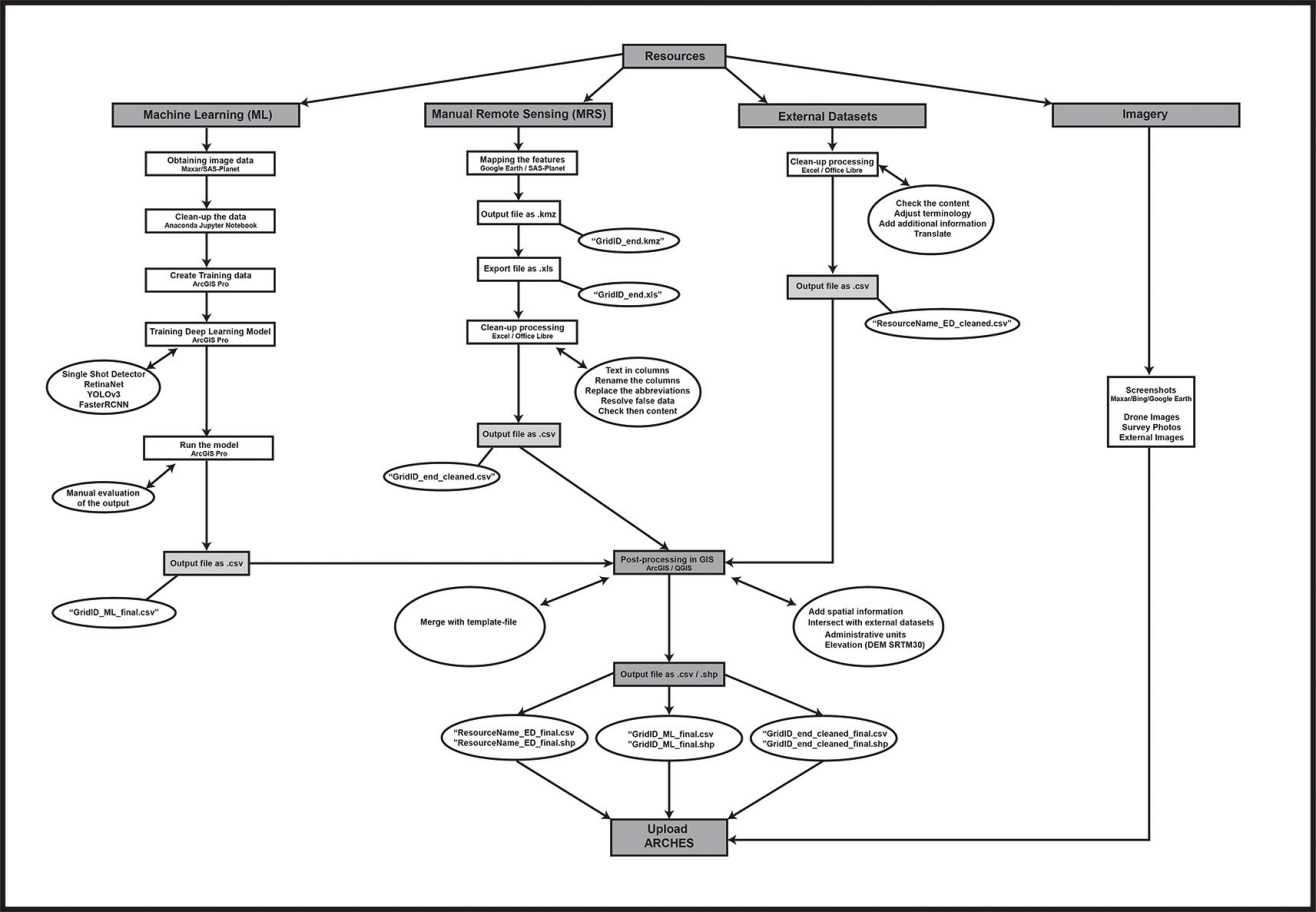
Diagram of the initial MAPSS workflow, integrating ML, MRS, external data, and imagery/field data.

The initial strategy to integrate the data resulting from these methodologies featured approximately parallel workflows for each of the three data types–external/archival datasets, MRS data, and DL-generated data–with integration between them achieved firstly in ArcGIS and then the Arches database via.CSV import ( Figure 3). MAPSS analysts use these geospatial data management applications to visualise and calculate convergences between the methods, achieving data integration through both applications but using each toward different ends.

**Figure 4.**
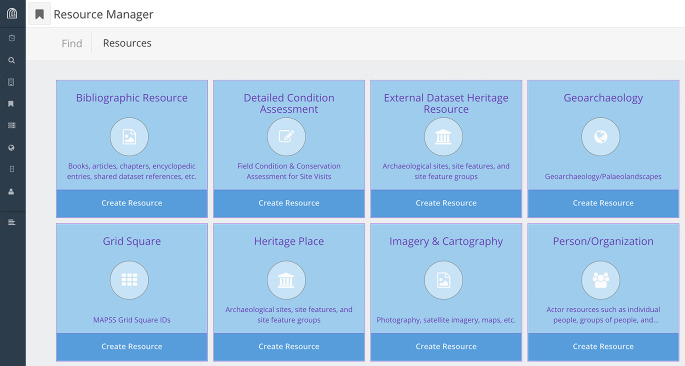
MAPSSdb Resource Manager in Arches showing the various Resource Models that house the graph-based data structures.

The Arches database will be the main access point to MAPSS data for the public and is the semantic heart of the recording system that integrates and informs the other components. Arches is a web-based geospatial semantic graph database platform that allows users to design fully customised, triple-store (RDF ^3^) graphs to structure data, and is CIDOC-CRM ^4^ compatible to ensure semantic interoperability ( Meyers 2016). The basic design of the MAPSS database comprises multiple resource models, each custom designed for specific purposes ( Figure 4), which are integrated through ‘resource-instance’ data-type nodes that are semantically embedded within and distributed throughout each graph.

**Figure 5.**
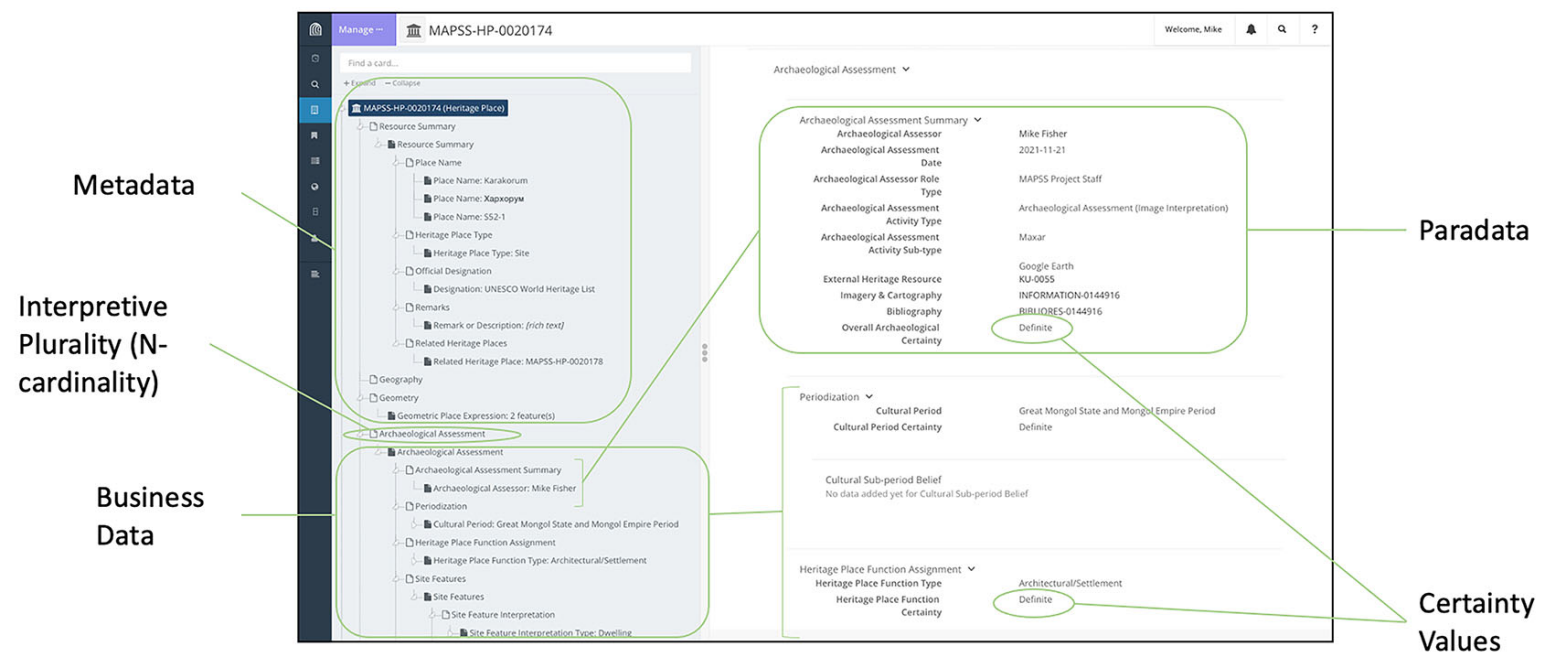
Diagram showing conceptual elements of a sample record in the MAPSSdb on the Arches platform.

The graph of each resource model utilises hierarchical data structures to capture multiplicity of interpretations, each one replete with its own metadata (including date, assessor name, and assessor affiliation) and paradata (including interpretation method and information resources used; see Matskevich & Sharon 2015: 107 ff.). The MAPSS graphs additionally model interpretive uncertainty throughout the graph design, allowing for greater reusability (the ‘R’ in the FAIR principles; Figure 5). Overall, this design follows the principles of ‘relativistic recording’, as opposed to determinist, positivistic recording (ibid.: 108).

Speaking further to the issue of reusability and sustainability of datasets, MAPSS has collaborated with developers and cultural heritage colleagues to redevelop the export and import functionalities of the Arches platform so as to improve interoperability across the divide between graph-structured databases and flat files. Most graph-based data do not fit flat-file formats such as.CSV or.SHP due, primarily, to their potential for N-cardinality both horizontally and vertically (i.e., N-children each with N-children). This type of cardinality flexibility, natively deployable in graph structures, is essential for modelling complex concepts such as interpretive plurality and uncertainty at scale. But because Arches at its core is, uniquely, a graph interfacing layer overlying a PostgreSQL relational-database backend ( Barton *et al.* 2017), it enables both importing data from and exporting data into SQL-like shapes across spreadsheets, accurately capturing the semantic logic and graph structure in spreadsheet format with zero data loss or ambiguity.

Upon learning of the plans for the construction of the Erdeneburen Dam and the accompanying archaeological and cultural surveys, MAPSS analysts processed archival datasets containing the field survey data that had already been generated by local entities such as the Khovd University Department of Archaeology. They then investigated the area using high-resolution satellite imagery, to confirm and further identify visible archaeological features and their condition states. Although threats and disturbances are less relevant for long-term heritage management of sites facing imminent inundation, as is the case within the Erdeneburen Dam Project flood zone, MAPSS documents all visible condition states and risks, for scientific purposes.

**Figure 6.**
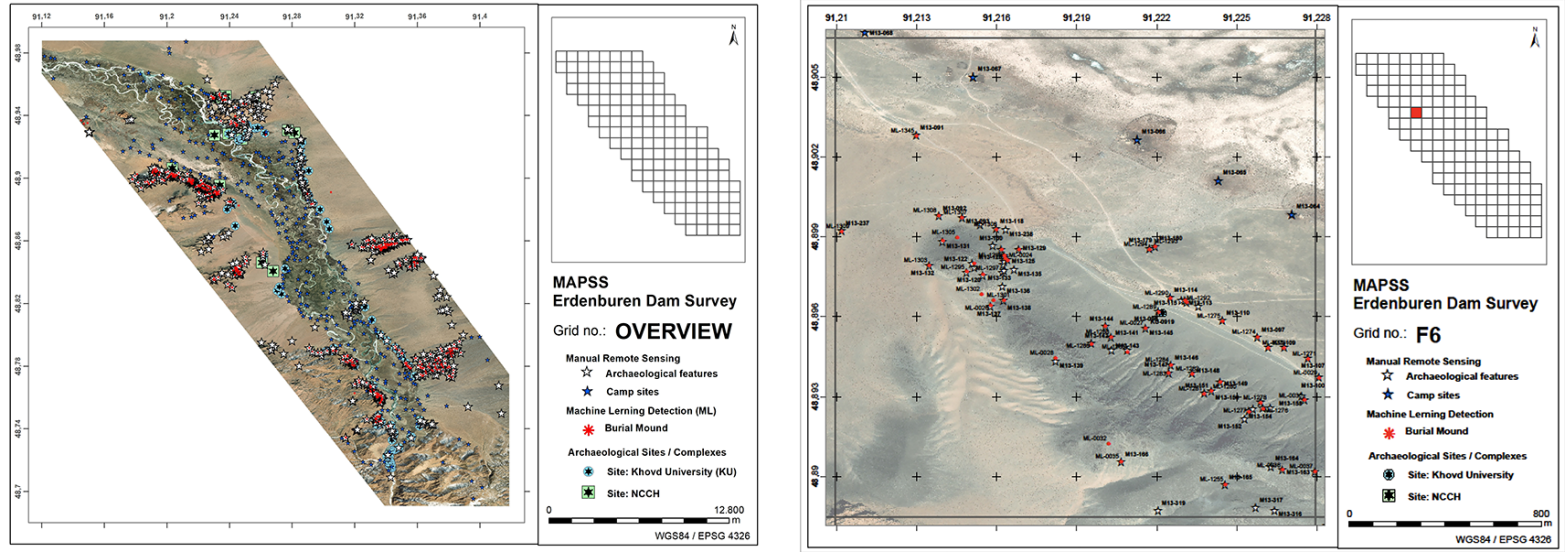
Sample pages of the MAPSS remote sensing portfolio for the Erdeneburen Dam area. Background satellite imagery source (open access): Maxar Technologies.

For local professionals in Mongolia, MAPSS is planning future training workshops to transfer the skills required to extract meaningful datasets from the Arches database. Due to the urgency of the Erdeneburen Dam Survey, in this case the project plotted the geospatial data over maps in ArcGIS to integrate the various datasets. The output was a printable portfolio, each page of which displayed a localised grid square illustrating feature locations ( Figure 6), that accompanied a.SHP file transferred to the Institute of Archaeology in advance of their survey season. Upon arrival at the Institute of Archaeology camp in Khovd, MAPSS personnel transferred the coordinates of archaeological features to partners’ GPS devices directly.

As the Mongolian government funded the Institute of Archaeology salvage expedition to target the flood and surrounding development zones exclusively, the MAPSS team focused on adjacent areas as well, in order to capture field data for assessment and monitoring of the archaeological sites that would remain above water. Simultaneously, MAPSS collected field data to evaluate remote sensing accuracy. In order to target certain high-density heritage areas, and expand the extent of the survey beyond the flood and development zones, the MAPSS pedestrian survey focused on all visible archaeological remains, mainly detecting four different feature types: funerary monuments such as *kurgans,* ritual monuments such as standing stones and pavements, ethnoarchaeological settlements such as prolonged-used winter camps, and rock art/cave sites.

**Figure 7.**
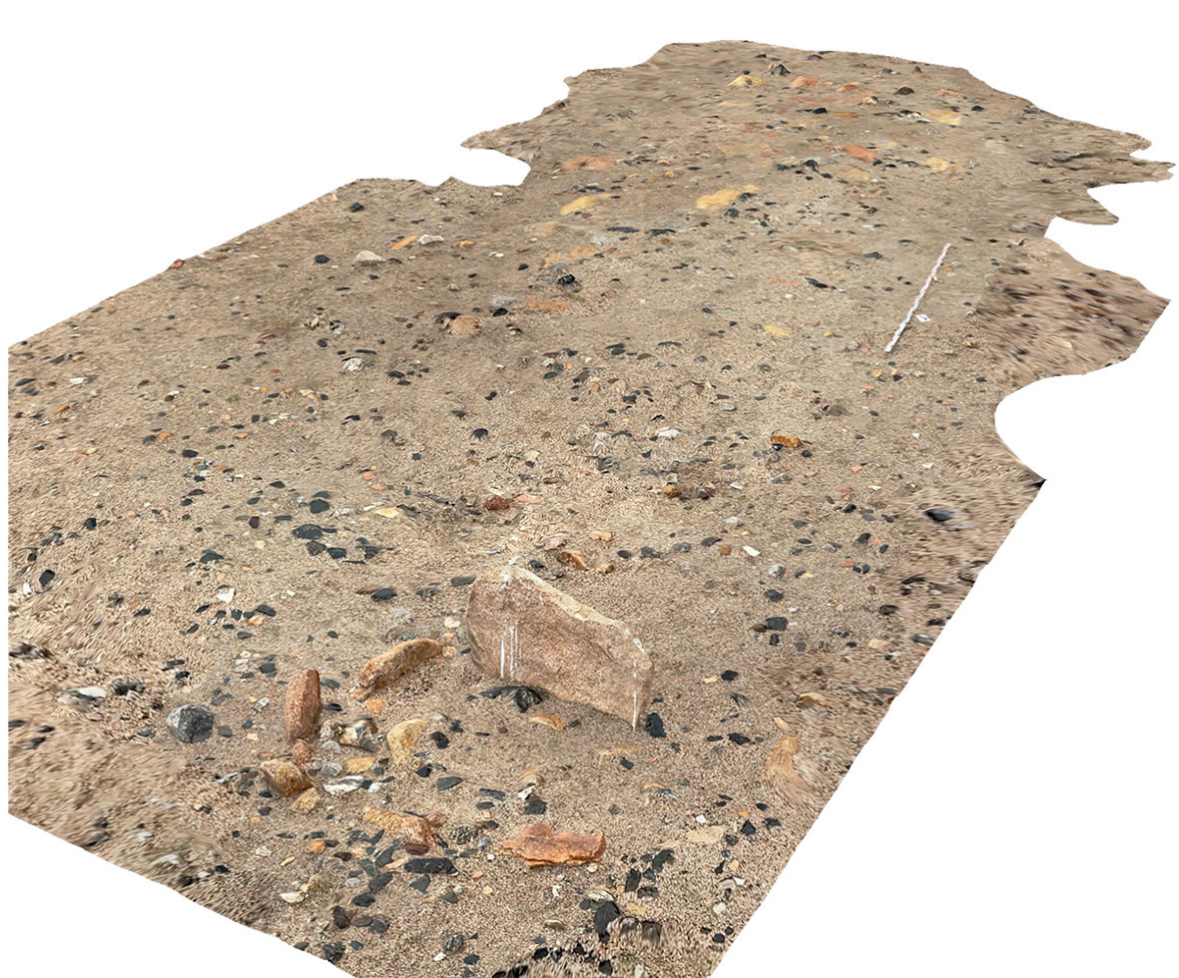
3D model depicting a ritual feature typical of the 2 ^nd^ mil. BCE, to be submerged.

The two aims were to ground-truth remotely sensed archaeological features and to record features unrecognisable in the open-acceess satellite imagery available for remote sensing. The survey participants included MAPSS team members based at the Max Planck Institute in Jena, Germany, and two partners based in Ulaanbaatar, Mongolia, Dr. Khurelsukh Sosorbaram and Batchuluun Oyundelger. Participants used a digital recording system for pedestrian survey that is designed to generate Arches data directly in the field, in addition to 2D visual recording with digital cameras and 3D visual recording using iPad LiDAR sensors (for a subset of archaeological features; Figure 7). Project members recorded survey data directly into spreadsheets on computer tablets, which can then be uploaded into Arches with minimal transformation and cleaning.

**Figure 8.**
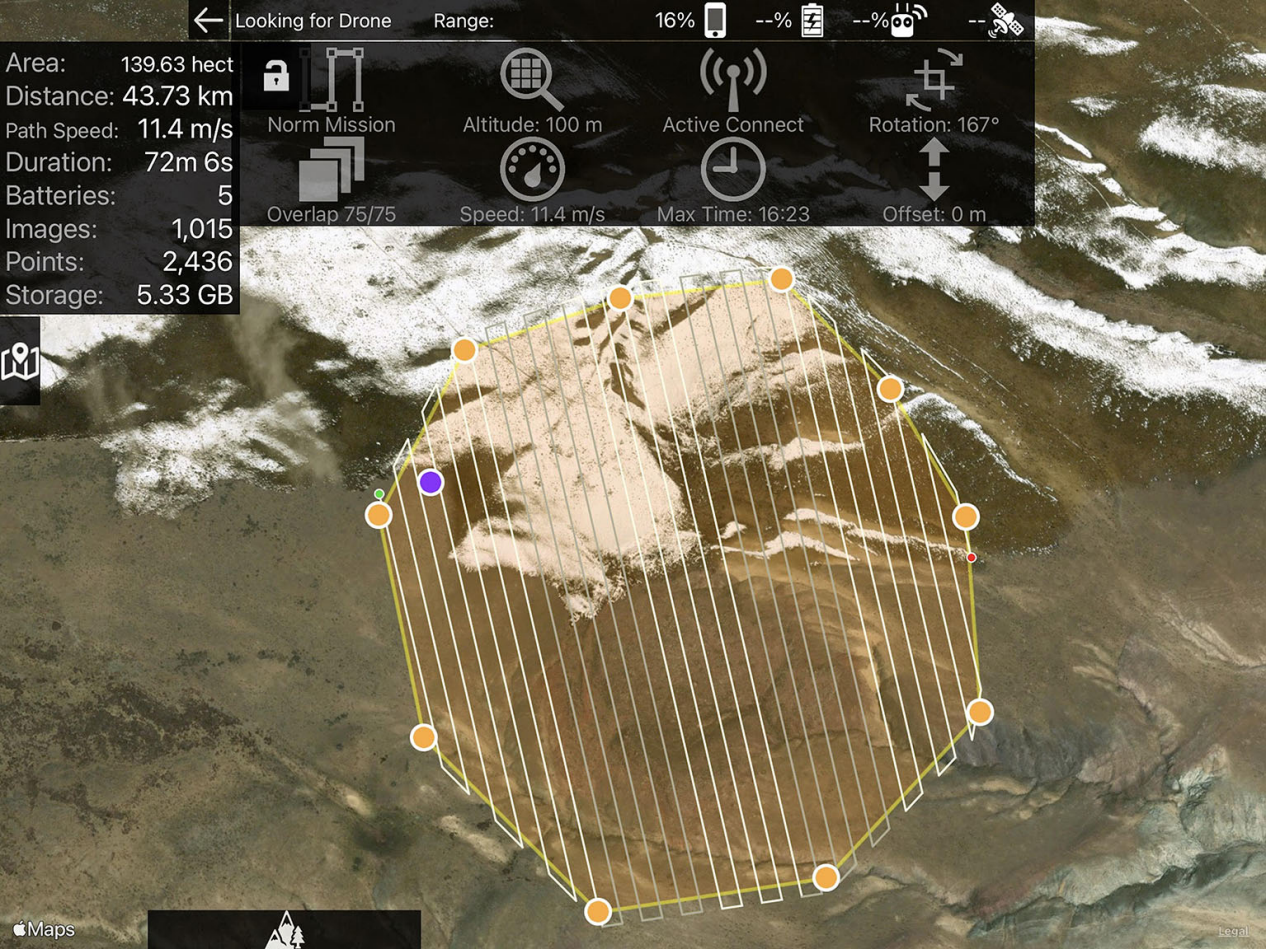
UAV flight plan in Map Pilot Pro from the MAPSS survey in Khovd.

An essential component of the MAPSS field methodology is using programmable, rotary-wing unmanned aerial vehicle (UAV) photography to generate Very-High-Resolution orthomosaics of the landscape, beneficial for remote survey of mountainous regions poorly covered by high-resolution satellite imagery ( Rouse & Krumnow 2020). The process begins by creating a flight plan in Map Pilot Pro, flying the drone over the selected area ( Figure 8), and then processing the images in either ArcGIS Pro or Agisoft Metashape. Georectification of the resulting image files enables the production of orthomosaics, Digital Surface Models (DSM), and other outputs.

**Figure 9.**
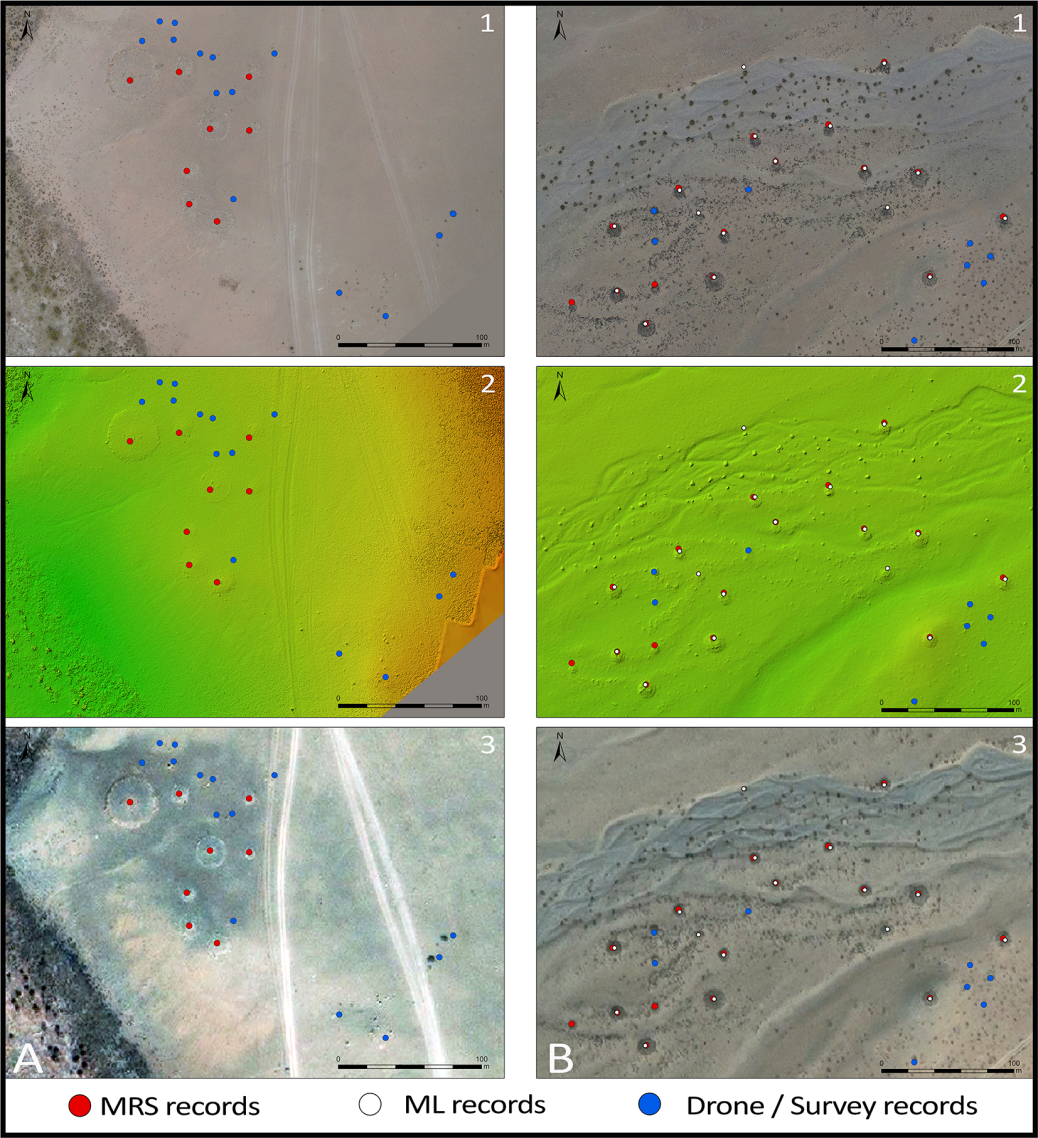
Imagery comparison. 1: drone-based orthomosaic; 2: drone-based DTM; 3: open-access satellite imagery (source: Maxar).

The resulting Very-High-Resolution imagery of the research areas allows MAPSS analysts to “drone truth” remote sensing data on a much broader scale than is possible through pedestrian survey alone, but with better spatial resolution than available commercial imagery ( Figure 9). For example, the MAPSS pedestrian survey covered approximately 1 km ^2^ in the Khovd River Valley research area, compared with 7 km ^2^ covered by drone mapping. With a typically 0.3m spatial resolution, the orthomosaics allow for effective reanalysis of targeted areas, clearly showing details unrecognisable in 1m-resolution imagery, including sub-3m objects such as standing stones as well as nearly flat-lying features such as stone pavements.

A main function of the MAPSS field survey activities (as opposed to the Institute of Archaeology survey) in the Khovd River Valley was to evaluate the efficacy of project methods such as remote-sensing site detection, as well as overall methodology. The testing targeted verification of the remote sensing results using pedestrian and drone mapping surveys, assessing confidence rates and reliability of the remotely sensed data. MAPSS randomly chose three sampling areas to survey across environmentally varied zones within the area of interest. The evaluation of the data is still ongoing, and so this paper will present the results of two sampling areas, Khovd Gol 1 and Khovd Gol 2. Simultaneously, the Erdeneburen Dam project provided MAPSS the opportunity to evaluate and test the broader methodological and theoretical frameworks in which it generates and curates its datasets.

## Results

**Figure 10.**
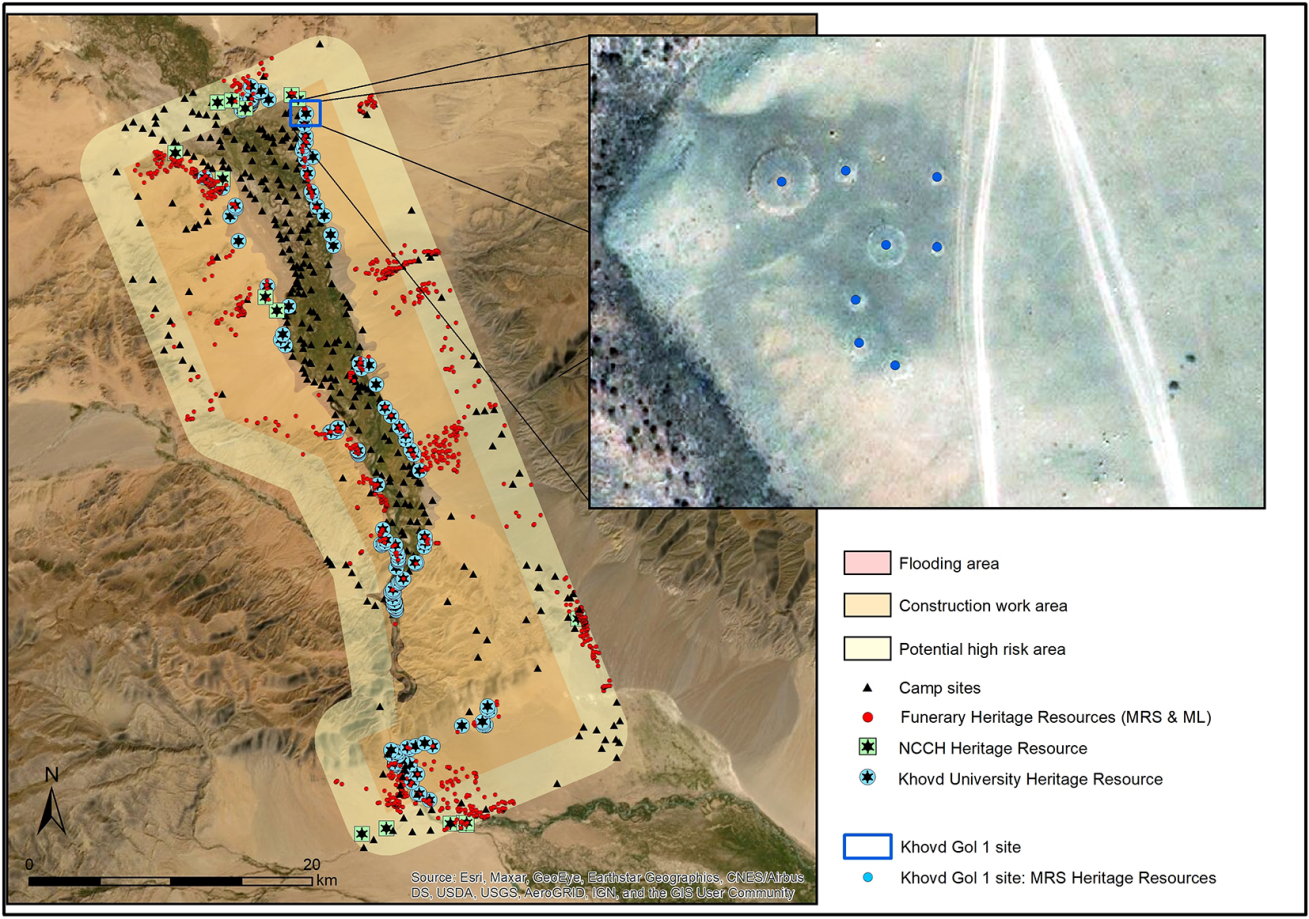
Erdeneburen Dam dataset shown across three activity zones. Khovd Gol 1 site in inset.

Satellite imagery of the flood zone and the broader area surrounding it suggests that the impact of the flooding will affect various areas of the valley differently, depending on proximity and topography. After georectifying the government maps that delineate the two impact zones, we then added a proposed extended monitoring zone of approximately 2 km ( Figure 10). The case study presented here includes two clusters of archaeological features that the project documented and analysed, one in the flood zone and the other in the extended monitoring zone.

The site of Khovd Gol 1, located inside of the flood zone, is situated on the slope or low cliff of the river valley ( Figure 10). The surrounding plain is steppe or semi-desert, presently containing almost no vegetation. All of the archaeological features documented have been exposed to natural depositional processes and many are at least partially covered by sand, rendering them unrecognisable in most types of remotely captured imagery.

**Figure 11.**
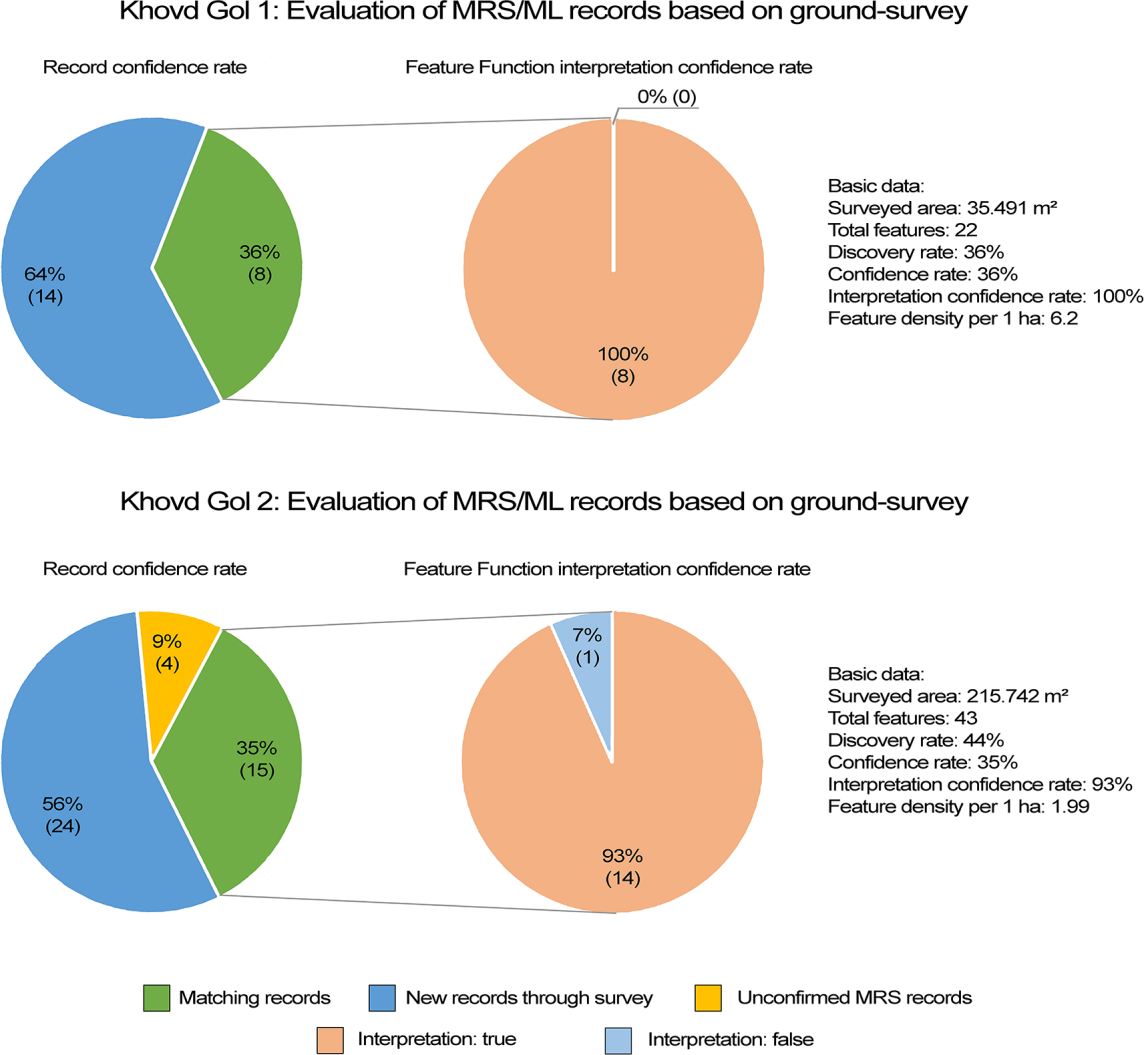
Confidence rates of remote-sensing feature detection based on ground truthing survey at two sites.

The pedestrian survey of Khovd Gol 1 recorded 22 features in total. Remote sensing had already identified eight features, while MAPSS identified only 14 (63.6%) in the field ( Figure 11). This indicates an approximate remote-sensing rate of 36%. The MAPSS survey achieved similar results at the Khovd Gol 2 site, which is situated in a rocky environment outside of the highest-risk areas. The pedestrian survey there recorded 43 features in total, including 15 (~35%) that analysts had already captured through remote sensing and 24 (~56%) discovered only through ground truthing (the remaining four were unverified by ground truthing).

A more complex evaluation comparing the total MRS data from the Khovd River Valley with external survey datasets addresses the different levels of detail recorded in each. Both the Khovd University (KU) dataset and the NCCH dataset mainly consist of “site” records, meaning one record represents multiple archaeological features, rather than representing individual features as MAPSS records do. Comparing MAPSS MRS with the more detailed, ground-based KU survey data, and considering only funerary monuments, 27% of the 199 sites appear in both datasets. This relatively low percentage of overlapping site identification is largely due to the KU pedestrian-survey recording of features too small to detect using freely available, open-access imagery. Alternatively, comparing the singular records within the surveyed area, the numbers approach parity: 300 funerary monuments recorded by the KU survey compare against 196 records generated remotely by MAPSS. According to our interpretation of the KU survey extent, 70 MRS records do not appear in the KU dataset. The divergences in each case suggest that both MRS and pedestrian survey have the potential to identify archaeological features that the other method might miss.

A different picture emerges by comparing MRS records with NCCH data. In the Khovd River Valley research area it is possible to correlate seven out of eight MRS sites with NCCH records. In other words, using remote sensing techniques, MAPSS identified 87% of the sites recorded by NCCH during pedestrian survey. Transposing these data to individual feature records, MRS identified 252 features compared with 168 features (from eight sites) that the NCCH had already recorded. MRS enabled MAPSS analysts to assign precise locations to most of the NCCH records, while also complementing the existing records with new discoveries. Comparison across all datasets shows a 63.3% overlap (660 records) between pedestrian survey in general and MRS data.

To summarise, MAPSS analysts identified approximately one-third of known features using MRS techniques. The majority of the features (56-65%) were field discoveries (i.e., not detected by remote sensing methods), due primarily to their small size. According to a preliminary assessment, the average approximate diameter of features unrecognised through remote-sensing methods is 3m, and does not exceed 8m, or otherwise are shallow/paved features. Though the evaluation of the results is ongoing, the ground-truthing based confidence in the features identified through MRS in Khovd Gol 1 and 2 is preliminarily at 90%.

**Figure 12.**
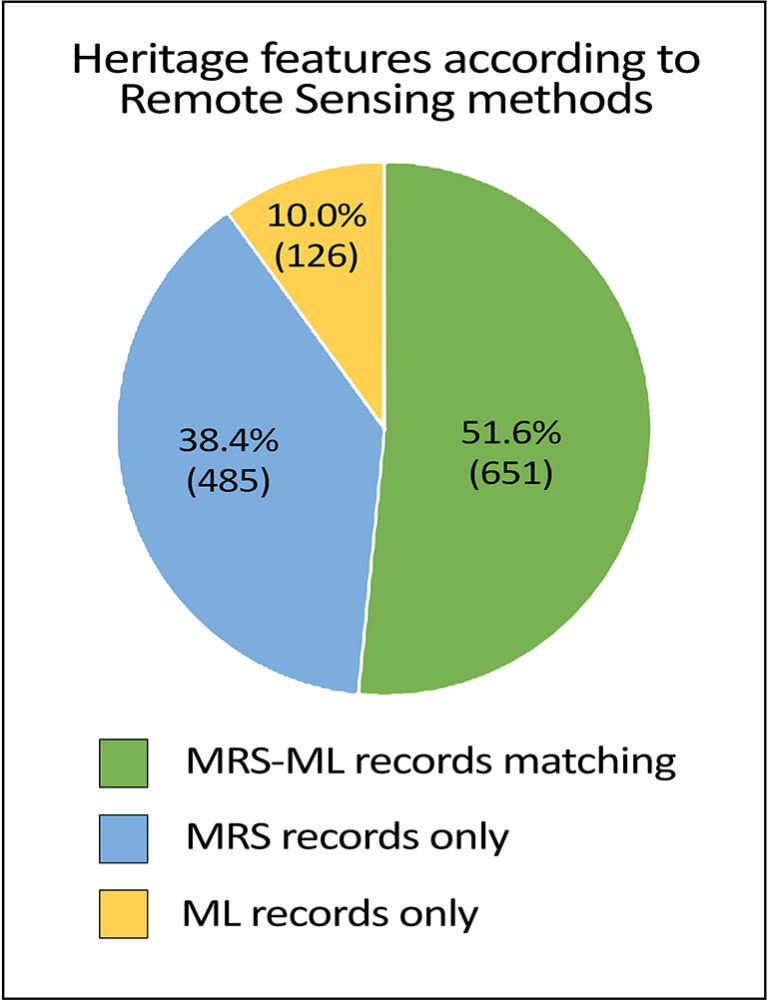
Contribution of each remote sensing method in detecting 1,262 Heritage Places in the research area.

We also compare ML/DL outcomes to MRS ( Figure 12). Approximately 10% (126/1262) of the records are exclusive to DL automated site detection. Based on raw numbers, DL detects 25.4% (777/1042) fewer archaeological features (e.g., individual burials or monuments) than MRS detection of features. However, DL detects a similar rate of the major clusters and sites, and so MAPSS ultimately factored this aspect of the different methods into its revised workflow and data integration strategy (see below).

For condition state, threat, and risk analyses, project members identified the threats and disturbances impacting heritage resources in the development and extended monitoring zones, including infrastructure, looting, and erosion. It is unfortunately a near inevitability that sites within the flood zone face complete submersion. The government-use zone represents an area subject to industrial and administrative activities necessary for constructing and operating the dam, and so has the potential for a very high rate of disturbance. The proposed extended monitoring zone will presumably incur a significant amount of population displacement from the other zones, although redefining this zone using demographic data and geographic analysis would be beneficial. Nevertheless, the simply defined area shown here will include not only potentially higher population density in places, but more destructively, new infrastructural development.

**Figure 13.**
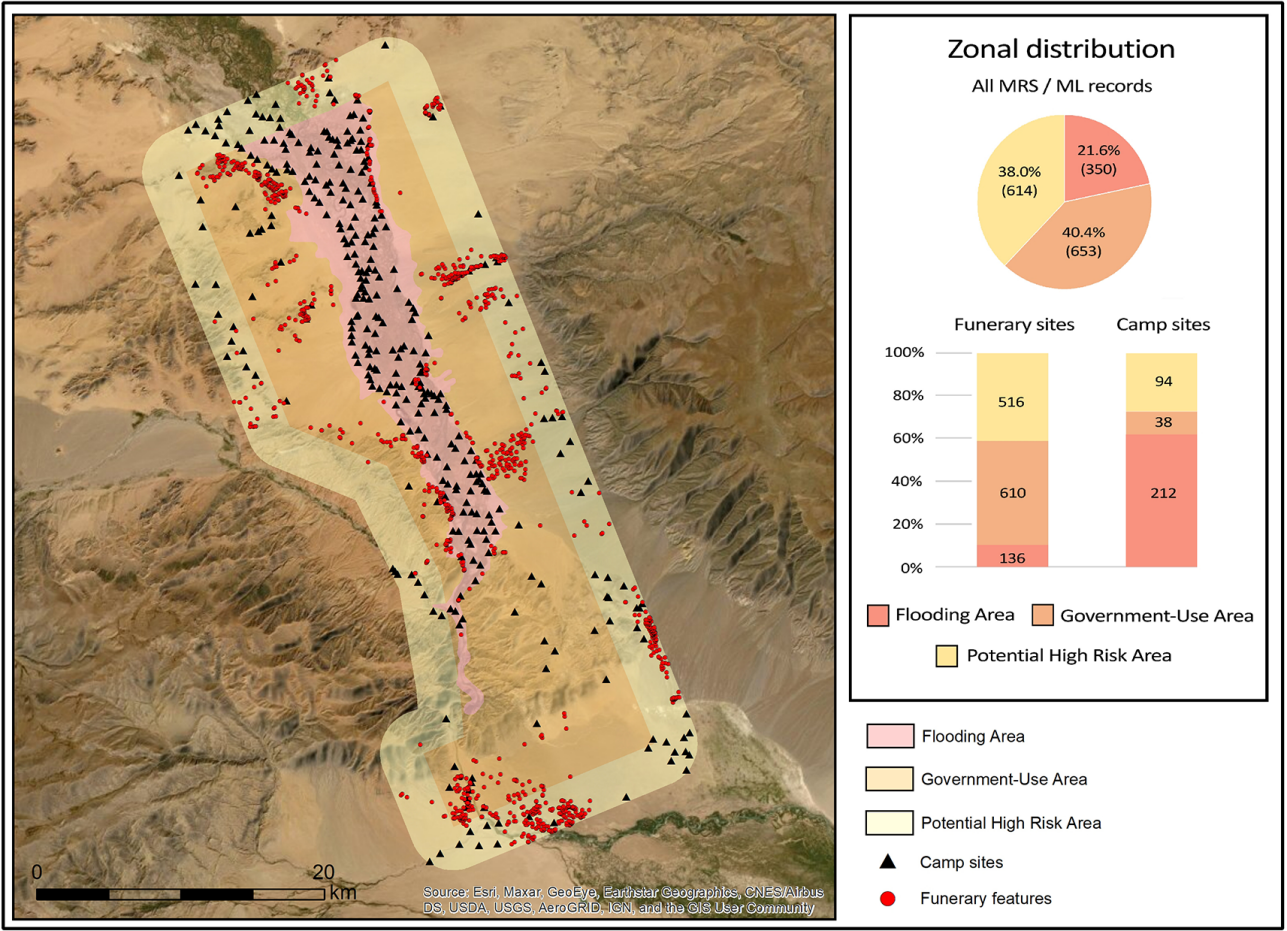
Distribution of Heritage Resources across the three endangerment zones of the Erdeneburen Dam Project.

According to the zonal distribution ( Figure 13), 21.6% of the recorded sites fall within the flood zone, although nearly two-thirds of those are campsites. Recording occurrences of seasonal habitation sites is primarily ethnographic in nature, although in many cases campsites evidence occupations dating to mediaeval ( Wright 2016) and prehistoric ( Houle 2016) periods. Clearly, the largest impact of the dam construction will be on the lives of the valley inhabitants, who will at some point be unable to return to their riparian spring-season camp locales. Over 600 ancient funerary monuments, however, will be subjected to potentially destructive activities carried out in the government-use zone, indicating that authorities and cultural heritage institutions should pay close attention to changing condition states of the archaeological features located there. Furthermore, the extended ‘possible high-risk’ zone contains hundreds of archaeological features as well as nearly one hundred campsites whose inhabitants will likely witness added population pressure and increased infrastructural development in their seasonal habitation areas.

The concentration of 610 (likely) ancient funerary sites in the government-use zone (out of 1,126 total) indicates that the destruction of access to cultural heritage effected by the dam construction can either expand–if sites are not monitored and protected–or be stemmed, through ongoing intervention efforts. A responsive/proactive mode of intervention would include measures that Mongolian authorities and their partners have already taken such as documentation and development of monitoring techniques. It would also require, of course, use of the resulting datasets and techniques to regularly assess the impact of dam-related processes on cultural heritage features and possibly deteriorating feature conditions.

**Figure 14.**
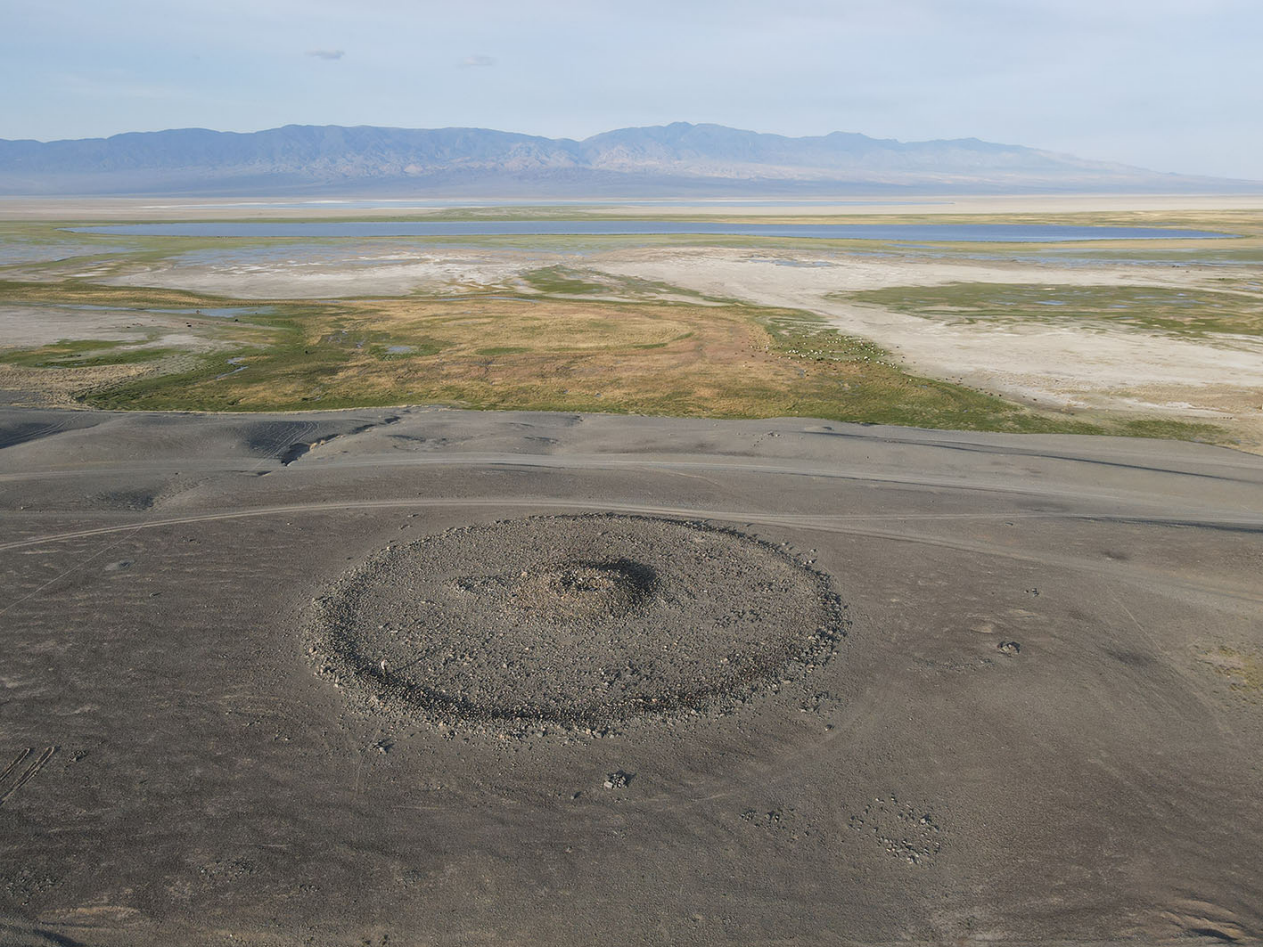
Drone image of a *khirigsuur* burial, typical of the 2nd millennium BCE, to be submerged.

MAPSS Khovd River datasets provide a basic foundation of information about the number and type of features that the dam construction will submerge and what will survive but be exposed to increased threats from associated development activities. This can facilitate strategic decision making for damage mitigation, rescue survey, and monitoring of heritage resources. Remote sensing methodology has an advantage over classical survey in cases such as these in terms of coverage area, time requirement, and human resource expenditure. Additionally, this case study provides the preliminary statistical evaluation of the MAPSS data and their confidence values, and so the methods complement one another. For example, areas with high occurrences of complex *khirigsuur* burials ( Figure 14) also feature higher (ground) detection rates of objects that are “invisible” to remote sensing, such as paved ritual features, than the areas featuring primarily simple stone mounds, which show higher rates of other object types such as standings stones. Enabled by the pedestrian and drone-based ground-truthing surveys, future analysis can evaluate whether there is any statistical correlation between set types of archaeological features.

## Discussion

The major outcomes of this case study have been: 1) the expansion of the surveyed zone, extending the monitoring capabilities beyond the government’s originally funded plan and demonstrating the benefits of cooperation between local and non-local entities; and 2) the feedback received on the shared dataset in combination with a self-evaluation of the MAPSS methodology, integration strategy, and data transmission pipeline.

Informal partner feedback (through unsolicited conversation) and project self-assessment, identifying a lower DL object-detection rate than MRS, both indicated that the MAPSS project needed to devise a stronger, more integrational methodology across the various fields of practice, which include cultural heritage preservation, field archaeology, geography, and computer science. In order to theorise such a strategy, this paper borrows the term ‘transmethodology’ from Khwaja and Kousholt, who describe it as a

… focus on phenomena through multiple theoretical perspectives and multiple interacting/connected empirical methods. This may also indicate a combination of qualitative and quantitative elements of inquiry and the creation of new onto-epistemological approaches and ethics ( Khwaja & Kousholt 2021: 3).

We consider this in conjunction with Symmetrical Archaeology, which Shanks (2007) defines as an “attitude” that removes the assumptive distinction between people, processes, and material remains. In a sense, this dovetails with the supposition that digital archaeology dematerialises the past ( Brouwer & Mulder 2003: 4; cf. Sluis 2017: 28). However, within a Symmetrical Archaeology rubric, the degree of materiality is less germane than the resulting impact of identifying, structuring, generating, and using those data ( Fisher *et al.* 2021). Finally, within this framework, the MAPSS project seeks to help develop communities of local digital archaeology and heritage specialists in order to practise ‘responsive’ and ‘proactive’ heritage interventions through Resilience Humanitarianism ( Hilhorst 2018), which identifies local communities as the primary heritage practitioners and the first emergency responders to heritage disasters.

So how does one operationalise these theoretical frameworks, thereby reifying them ( Shanks & Tilley 1992: 49)? As a digital documentation project tasked with identifying and recording as much of the immovable cultural heritage of Mongolia as possible, any methodology that MAPSS applies has to remain focused on productivity and quantifiable results, while still realising that there is more to productivity than the technical processes.

**Figure 15.**
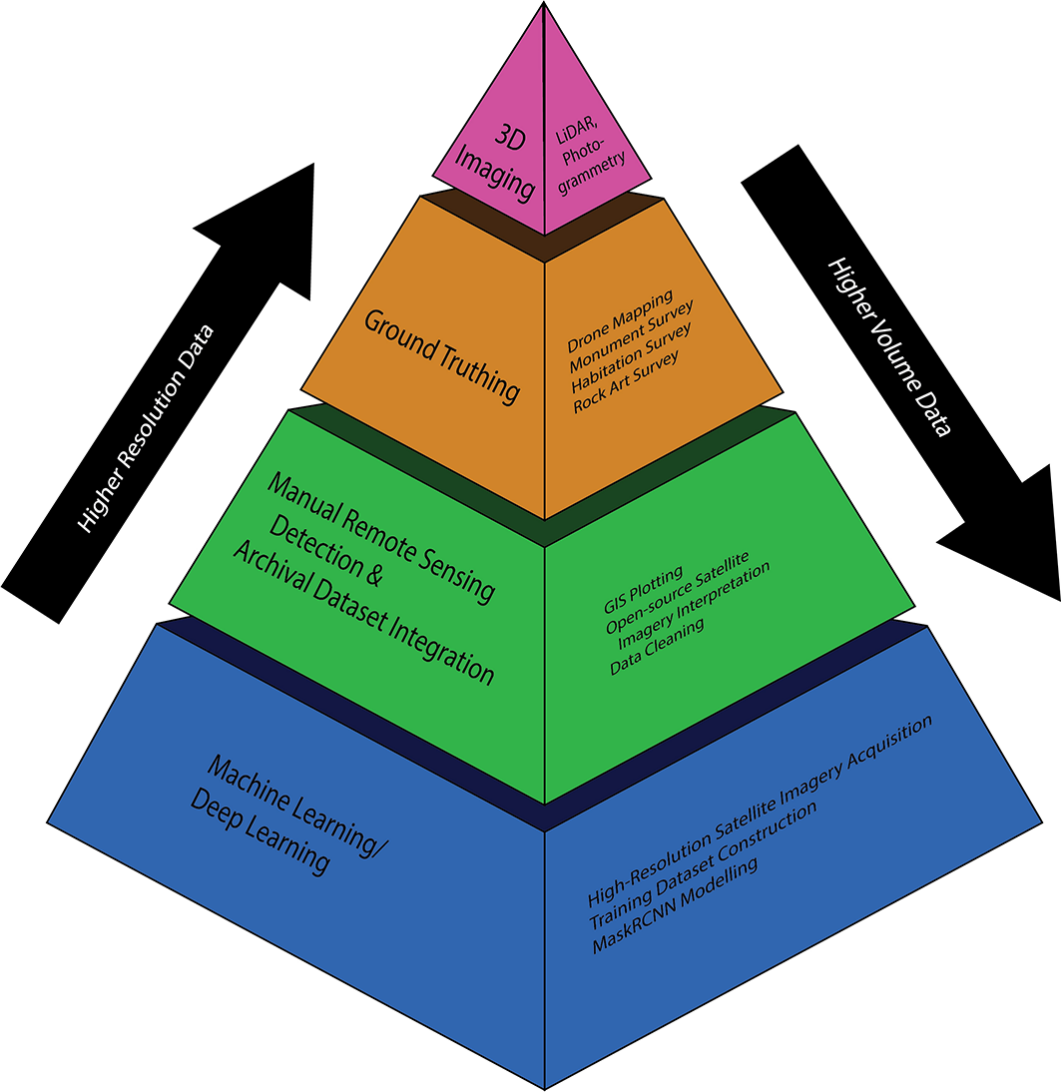
MAPSS site detection and recording methodological pyramid.

The first step is to integrate the various areas of practice into a single, principled methodology in which recording moves from higher-volume/lower-resolution sensing capabilities to lower-volume/higher-resolution sensing capabilities, with each scalar informing the next ( Figure 15). DL-automated remote sensing now provides the project’s baseline of sites, from which it is then possible to decide on areas for further investigation through MRS. This adds a higher degree of certainty, greater number of feature detections, and richer interpretations and assessments, but over a more limited spatial extent. From the MRS dataset, the MAPSS project can select research areas for field-based drone mapping ( Hritz 2010), from which analysts generate very-high-resolution orthomosaic imagery. Within each UAV fly zone, the project can then choose smaller areas for pedestrian survey, including investigation of potential rock-art sites, based on local guidance. Within each of those pedestrian survey zones, survey participants then select a subset of monuments for 3D scanning, primarily using hand-held LiDAR sensors equipped on iPad devices.

**Figure 16.**
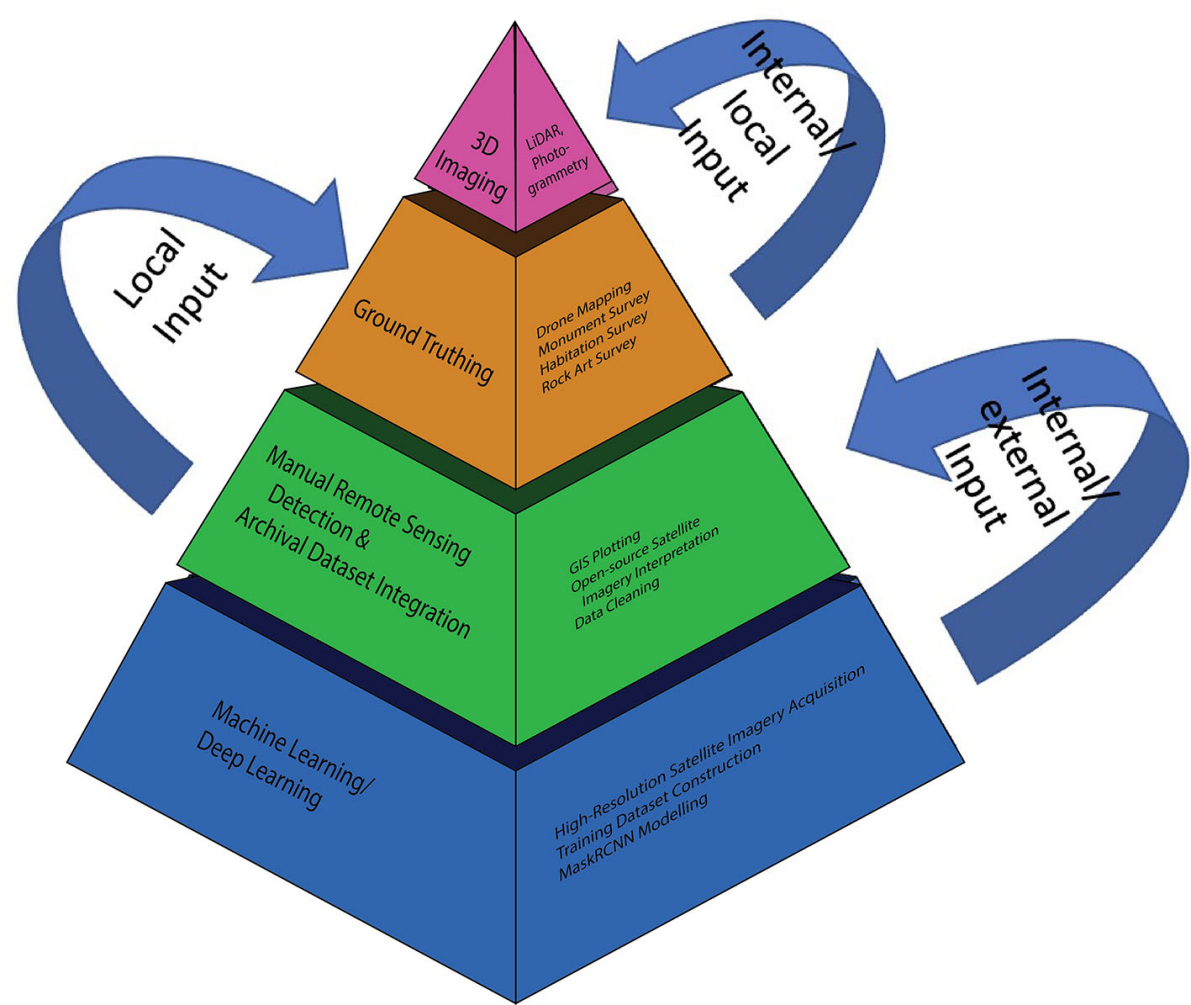
Input points within the MAPSS methodology.

**Figure 17.**
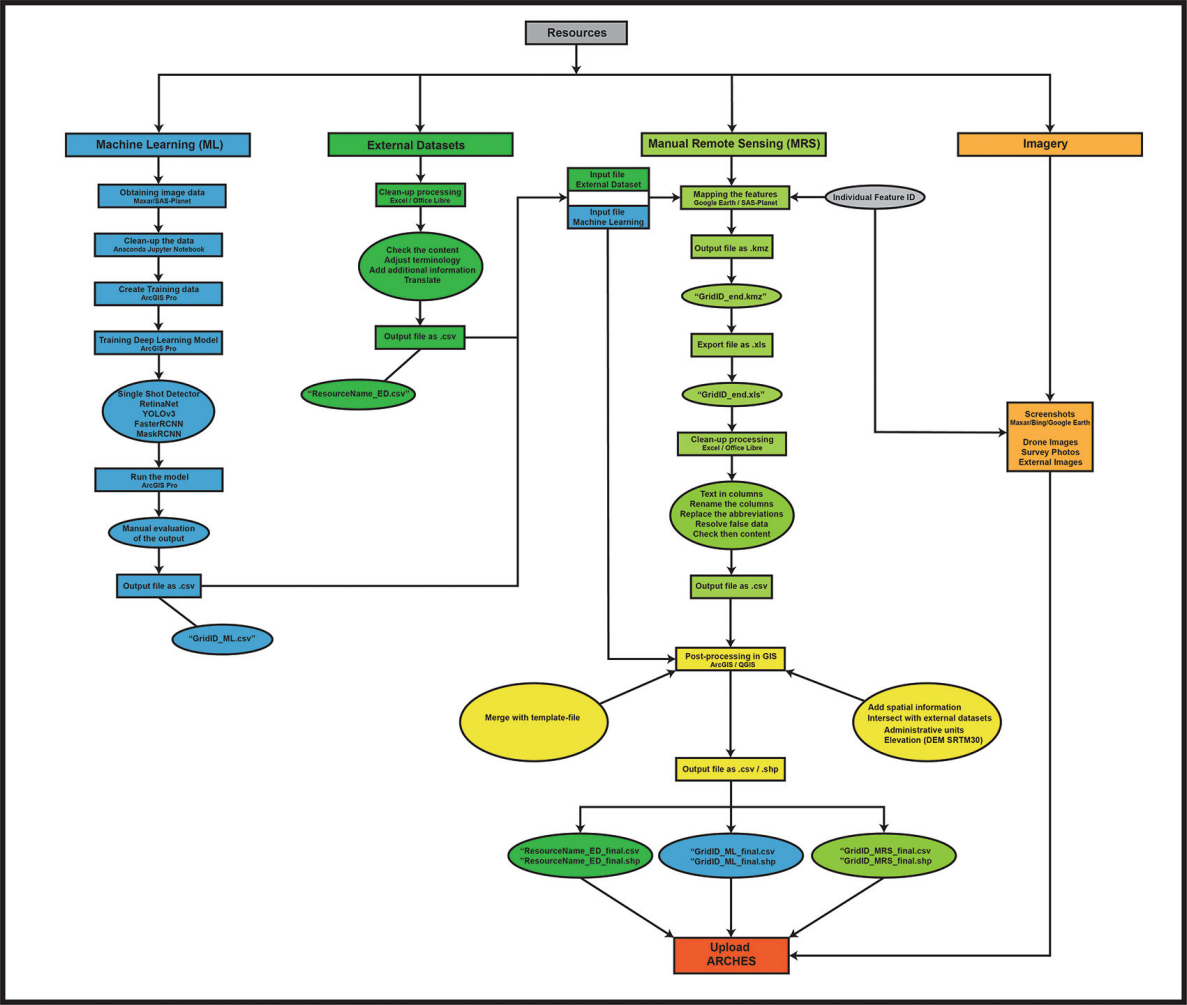
Revised MAPSS workflow, with colours (blue, green, and orange) corresponding to scalars in Figures 15, 16.

Each scalar in the pyramid requires not only internal evaluation for informing the next scalar, but in order to adhere to principles of Symmetrical Archaeology, Resilience Humanitarianism, and the CARE principles, external input from local archaeological and cultural heritage communities as well ( Figure 16). A revised workflow diagram illustrates this, demonstrating how the new MAPSS ‘transmethodology’ functions in practice ( Figure 17). The colours of each action node correspond to their scalar in the methodology pyramid.

In conclusion, the MAPSS project, in collaboration with the Institute of Archaeology, assisted with a ‘responsive intervention’ approach to the Khovd River Dam emergency. It is ‘responsive’ rather than ‘reactive’ because, although triggered by a punctuated emergency, it in-builds the potential for not only better monitoring of affected (but above-water) sites, but also a broader methodology that improves both input feedback and output data. This provides more optimal conditions for ‘proactive’ heritage preservation efforts, both in relation to the Erdeneburen Dam construction zone and across Mongolia more broadly. The MAPSS component of this international, trans-institutional collaboration has also produced statistical analysis of its remote discovery methods, identified an expanded set of endangered archaeological sites, and incorporated local partner feedback to re-evaluate how it integrates the various methodologies into a single, productive, and principled transmethdology that balances output with impact.

## Software availability

Software available from: https://github.com/orgs/MPI-MAPSS/repositories

Source code available from: https://github.com/MPI-MAPSS/, https://github.com/MPI-MAPSS/MAPSSdb

Archived source code at time of publication: https://doi.org/10.5281/zenodo.7188788 ( Fisher 2022).

License: Creative Commons Zero v1.0 Universal, GNU Affero General Public License v3.0

## Data Availability

All archaeological business data is deposited at https://mapss.server.shh.mpg.de/ and will be made available to the public as of 01.12.2022. Due to the sensitive nature of geospatial data (i.e., site locations), and ethical considerations such as local authority to control, data access requests will have to be approved upon submission of name, valid email address, intent of use, and professional affiliation(s). Before 01.12.2022, data is available upon request via email to mapps@shh.mpg.de.

## Footnotes

^1^
The FAIR principles state that data should be Findable, Accessible, Interoperable, and Reusable ( Wilkinson *et al.* 2016; Letellier *et al.* 2007; Quintero *et al.* 2020).

^2^
The CARE principles state that data should prioritise Collective benefit, Authority to control, Responsibility, and Ethics.

^3^
A Resource Description Framework (RDF) is a semantic protocol that structures variables (fields) into “triple-stores” always featuring two nodes (variables) and an edge (relationship property) between them.

^4^
CIDOC-CRM is the Comité International pour la Documentation de l'ICOM Conceptual Reference Model
.
