# Supplementary material for: Multidisciplinary digital methodologies for documentation and preservation of immovable Archaeological heritage in the Khovd River Valley, Western Mongolia
Source: F1000Res. 2022 Nov 3;11:1250. [Version 1] doi: 10.12688/f1000research.126740.1 (PMC11387942; doi:10.12688/f1000research.126740.1)
Supplement: Peer review history

# Reviewer report: Reviewer response for version 1

**DOI:** 10.5256/f1000research.139178.r174172

**Published:** 2024-09-10

**Authors:** Fanar Mansour Abed

**Affiliations:** 1 College of Engineering, University of Baghdad, Baghdad, Baghdad Governorate, Iraq

The research presents an approach to integrate noninvasive techniques using MRS methodologies with the field invasive discoveries to improve CH preservation in Mongolia. It also demonstrates the potential of the CV and DL algorithms to speed the digital process based on multiple training DL Modules.

 The article is well written and presented. The literature included are up to date, however it should include more studies regarding the discussion of DL module effectiveness in CH. The technical contents are valued and the methodology presented is well illustrated but further suggestions for future improvements are required including results outcomes and analysis. However, some details are missing regarding the integration of the recorded data from Apple Lidar and what outcomes has delivered from integrating range-based data with image-based data.

Future insights:

 Previous studies have claimed that standalone data are vital for prospecting archaeological features. However, combining multi-datasets derived from different RS sources is more effective in digital archaeology to reveal uncovered remains. For example, digital hybrid raster images can be extracted from the RS data combination approaches in order explore buried archaeology and not only confirm detected features through the RS standalone raster products, see ( https://www.mdpi.com/2072-4292/15/4/1057). These rasters can derive from SfM photogrammetry, LiDAR data, multispectral images, etc. those have different specifications (e.g., different cameras, sensors, spatial resolutions, and GSD).

Is the work clearly and accurately presented and does it cite the current literature?

Yes

If applicable, is the statistical analysis and its interpretation appropriate?

I cannot comment. A qualified statistician is required.

Are all the source data underlying the results available to ensure full reproducibility?

Yes

Is the study design appropriate and is the work technically sound?

Yes

Are the conclusions drawn adequately supported by the results?

Yes

Are sufficient details of methods and analysis provided to allow replication by others?

Partly

Reviewer Expertise:

Geospatial Engineering, CV Photogrammetry, Lidar

I confirm that I have read this submission and believe that I have an appropriate level of expertise to confirm that it is of an acceptable scientific standard.

## References

[1] : Combining Remote Sensing Approaches for Detecting Marks of Archaeological and Demolished Constructions in Cahokia’s Grand Plaza, Southwestern Illinois. *Remote Sensing* .2023;15(4) : 10.3390/rs15041057 10.3390/rs15041057

# Reviewer report: Reviewer response for version 1

**DOI:** 10.5256/f1000research.139178.r177079

**Published:** 2023-07-04

**Authors:** Cinzia Bettineschi

**Affiliations:** 1 University of Augsburg, Augsburg, Germany

The paper titled "Multidisciplinary digital methodologies for documentation and preservation of immovable Archaeological heritage in the Khovd River Valley, Western Mongolia" presents a relevant contribution to the disciplines of archaeology, RS, and heritage science. The authors have successfully documented and evaluated the (ethno)archaeological remains in different impact zones associated with the construction of the Erdeneburen Hydroelectric Dam in western Mongolia. Their work showcases how invaluable is the integration of various methodological approaches and emphasizes the urgent need for a 'responsive' mode of heritage disaster intervention.

 One of the strengths of this paper lies in its comprehensive methodology. By employing GIS, Machine Learning automated site detection, drone mapping, and Structure-from-Motion, the authors have compiled a rich dataset that provides crucial insights into the threatened archaeological heritage of Mongolia.

 The results presented in the paper are both informative and thought-provoking. It is commendable that approximately 20% of the documented sites fall within the planned flood zone, highlighting the immediate threats posed by the construction of the dam. Equally important is the acknowledgment of collateral threats, such as industrial and infrastructural development, which necessitate extended monitoring. This recognition underscores the authors' commitment to preserving not only the sites directly affected but also the broader archaeological landscape.

 I have particularly appreciated the relevance given to fieldwork as an irreplaceable mean for data acquisition, and the efforts in balancing remote (and sometimes automated) methods for fast landscape screenings with detailed ground surveys on well-selected areas.

 The theoretical framework adopted by the authors, the high ethical standards of the cooperation, and the specific attention to metadata and paradata, add depth to their work. By intertwining these principles, the authors establish a solid foundation for this methodology, emphasizing the collective benefit and responsibility of mapping, monitoring, managing and preserving cultural heritage. This inclusive approach ensures that multiple perspectives are considered, thus enhancing the overall effectiveness of heritage preservation efforts.

 The literature is abundant and well-selected. It reflects a comprehensive understanding of the subject matter, incorporating a wide range of scholarly sources from various disciplines relevant to the study.

 While the paper presents a comprehensive analysis, there are areas that could benefit from further improvement and clarification. Specifically, more information is needed regarding the implementation of the case study itself and certain points require clarification.

 Firstly, in the abstract, the authors mention “SfM Lidar scanning”. However, it is important to note that SfM (Structure-from-Motion) and Lidar (Light Detection and Ranging) are two distinct methods with different interpretative potential, especially in forested areas, and they require different processing strategies, even if they both produce point clouds. The authors should provide clarification on whether they used SfM, Lidar, or both in different contexts.

 Additionally, while the paper delves into the details of the software and algorithms used for their neural network (NN), it would be valuable to have more practical information related to the case study. Specifically, the paper mentions classes of mounds and winter-camps, but it appears that there may be additional features that could be discussed. Furthermore, details about the quality and quantity of training data used for each class, their homogeneity, and the validation strategy should be provided. It is crucial to explain how the confidence rate for the results was calculated. Moreover, the potential and limitations of the method, as well as its accuracy compared to traditional survey methods like MRS (Manual Remote Sensing) and pedestrian surveys, should be clearly described. This is because remote archaeological objects suffer from issues of equifinality and multifinality (see Magnini and Bettineschi, 2019 ^1^) which can create important bias in the results of the automated predictions, if not carefully taken into account.

 Regarding UAV surveys, it would be beneficial to clarify whether RTK (Real-Time Kinematic), PPK (Post-Processed Kinematic), or GCPs (Ground Control Points) were utilized and to provide information about the accuracy of the measurements.

 Moving on to some minor points:

 Figure 1: It would be helpful to add a scale bar to the figure on the left side to provide a clearer understanding of the sub-area's extent.

 Figure 6 (left): The grid in the figure does not indicate the location of the area, and it should be addressed.

 Figure 7: It would be advantageous to include an orthophoto or plan of the area to provide scale and context.

 Figure 8: Please add a scale and north arrow to aid in interpretation.

 Figure 9: Please include a north arrow for reference.

 Lastly, it is stated that the data should already be available to the public as of 01.12.2022, but the provided website ( https://mapss.server.shh.mpg.de/) is currently inaccessible. Please correct.

 In conclusion, while the paper offers a comprehensive analysis, addressing the points mentioned above and ensuring clarity in the practical details of the case study would enhance the overall quality and impact of the research.

Is the work clearly and accurately presented and does it cite the current literature?

Yes

If applicable, is the statistical analysis and its interpretation appropriate?

Partly

Are all the source data underlying the results available to ensure full reproducibility?

Partly

Is the study design appropriate and is the work technically sound?

Yes

Are the conclusions drawn adequately supported by the results?

Yes

Are sufficient details of methods and analysis provided to allow replication by others?

Partly

Reviewer Expertise:

Archaeological Remote Sensing

I confirm that I have read this submission and believe that I have an appropriate level of expertise to confirm that it is of an acceptable scientific standard, however I have significant reservations, as outlined above.

## References

[1] : Theory and practice for an object-based approach in archaeological remote sensing. *Journal of Archaeological Science* .2019;107: 10.1016/j.jas.2019.04.005 10-22 10.1016/j.jas.2019.04.005

# Reviewer report: Reviewer response for version 1

**DOI:** 10.5256/f1000research.139178.r177077

**Published:** 2023-06-23

**Authors:** Georgia Andreou

**Affiliations:** 1 University of Southampton, Southampton, England, UK; 2 Institute of Archaeology, University College London, London, England, UK

This paper presents the results of a cultural heritage documentation project in Mongolia. The results are the collaborative effort of the MASS project and the Mongolian Institute of Archaeology to document sites directly and indirectly impacted by an upcoming dam construction. The authors focus on  interdisciplinary and multiscalar data collection methodologies, highlight the conceptual model of the used database, and demonstrate the importance of data quality control. Overall it is a well-written paper that employs a thoughtful, robust and technically sound methodology to document archaeological sites. The clear presentation of their ground- and drone-verification methods are to be commented. 

 The work builds on appropriately cited extensive research on big geospatial datasets. The method and analyses are presented in sufficient detail, allowing for their replication.

 Though the paper provides a link for the source data ( https://mapss.server.shh.mpg.de/), which are mentioned to be available to the public as of 01/12/2022, that link is not functioning. This review was thus unable to assess the quality of the source data.

 Several aspects of the paper require either clarification or further detail. Specifically:

1. A brief description of the terms "ethnographic" and "ethnoarchaeological" sites. Are they distinguished from other archaeological sites due to their chronology?
2. The concept of symmetrical archaeology is mentioned briefly in the introduction, while further detail is provided in the discussion section. It would appear more useful if further detail on the meaning and deductive capacity of symmetrical archaeology was introduced earlier in the text.
3. Explanation of "attentive digital archaeology program".
4. Figure 2 presents deterioration as a major factor impacting cultural heritage in Mongolia. Assuming that there are no archaeological sites not experiencing deterioration, it would be useful (perhaps in the caption) to clarify if deterioration is particularly rapid or acute in Mongolia.
5. Provide further detail for a clearer distinction between proactive and responsive action. 
6. The paper mentions that the authors received constructive feedback from the Mongolian Institute of Archaeology. It would be  interesting to see what that feedback  was about, as it is coming from the perspective of those working on the ground.
7. The workflow figures are detailed and very useful. They would be easier to digest if they were more simplified (e.g. figure 3), perhaps with the removal of specific file names in the diagrams.
8. The paper identifies possible risks to heritage related to potential population increase and associated infrastructure. Considering the very low population density in Mongolia, is there evidence that the area under examination will experience population pressures in the foreseeable future?
9. Provide further detail for a clearer distinction between transmethodology and interdisciplinary methodology.

All these are minor issues and providing further detail/clarification would improve the already high quality of this paper.

Is the work clearly and accurately presented and does it cite the current literature?

Yes

If applicable, is the statistical analysis and its interpretation appropriate?

Yes

Are all the source data underlying the results available to ensure full reproducibility?

No

Is the study design appropriate and is the work technically sound?

Yes

Are the conclusions drawn adequately supported by the results?

Yes

Are sufficient details of methods and analysis provided to allow replication by others?

Yes

Reviewer Expertise:

Cultural heritage; heritage big data; Mediterranean Archaeology; Near Eastern Archaeology

I confirm that I have read this submission and believe that I have an appropriate level of expertise to confirm that it is of an acceptable scientific standard, however I have significant reservations, as outlined above.
